# Adrenal crisis occurring after the application of immune checkpoint inhibitors in a hepatitis B - related hepatocellular carcinoma patient: case report and literature review

**DOI:** 10.3389/fimmu.2025.1604740

**Published:** 2025-11-27

**Authors:** Yue Guo, Qiang Zhu, Wanhua Ren, Feifei Li

**Affiliations:** Department of Infectious Diseases, Shandong Provincial Hospital Affiliated to Shandong First Medical University, Jinan, Shandong, China

**Keywords:** hepatitis B - related hepatocellular carcinoma, immune checkpoint inhibitors, adrenal crisis, hypophysitis, adverse reactions

## Abstract

Immune checkpoint inhibitors (ICIs) have become an important part of the treatment for hepatocellular carcinoma (HCC). However, the immune - related adverse events (irAEs) induced by them have also received increasing attention. This case report describes a rare case of adrenal crisis that occurred in an HCC patient after receiving sintilimab combined with targeted therapy. The patient presented with fever, acute abdomen, blood glucose 2.79 mmol/L, blood sodium 130.9 mmol/L, ACTH <1.5 pg/mL, COR 5.16 μg/dL, and progressive hypotension occurred. As for the timely diagnosis and active rescue by the multidisciplinary team (MDT), especially the timely supplementation of glucocorticoids, the patient’s changes was effectively controlled. This is the first adrenal crisis case treated in our department. The successful management of this case emphasizes the importance of recognizing endocrine crises induced by ICIs. When diagnosing and treating liver cancer patients who have received targeted combined immunotherapy, it is necessary to distinguish between septic shock and adrenal crisis to avoid misdiagnosis or missed diagnosis.

## 1 Introduction

Hepatocellular carcinoma (HCC) is a malignant tumor with increasing morbidity and mortality worldwide (1). Immune checkpoint inhibitors (ICIs) show potential in the treatment of HCC, but their use may be accompanied by the risk of immune-related adverse events (irAEs). Studies have shown that HCC patients treated with ICIs have a similar overall incidence of irAEs but a higher risk of liver-specific irAEs compared to patients with other malignancies (2).

Adrenal crisis, also known as acute adrenal cortical insufficiency or Addison’s crisis, is a relatively rare type of immune - related adverse event (IrAE). Its clinical manifestations may include fever, anorexia, nausea, vomiting, abdominal pain, diarrhea, lethargy, tachycardia, hypotension, shock, and even coma. Laboratory tests often show reduced plasma cortisol levels, along with electrolyte abnormalities such as hypoglycemia, hyponatremia, and hyperkalemia. Timely recognition and treatment of adrenal crisis is crucial, otherwise it may seriously threaten the patient’s life (3).

This report presents a case of HCC patient with typical clinical manifestations of adrenal crisis after receiving targeted combined immunotherapy. Thanks to the timely diagnosis and active rescue efforts by the multidisciplinary team (MDT), especially the rapid supplementation of glucocorticoids, the patient’s condition was effectively controlled. This is the first case of adrenal crisis successfully treated in our department, and the successful management of this case highlights the importance of recognizing endocrine crisis induced by ICIs. In the diagnosis of hepatocellular carcinoma patients receiving targeted combined immunotherapy, special attention should be paid to identify septic shock and adrenal crisis in order to avoid misdiagnosis or omission of diagnosis.

### 1.1 Patient information and admission history

**Figure 1:**
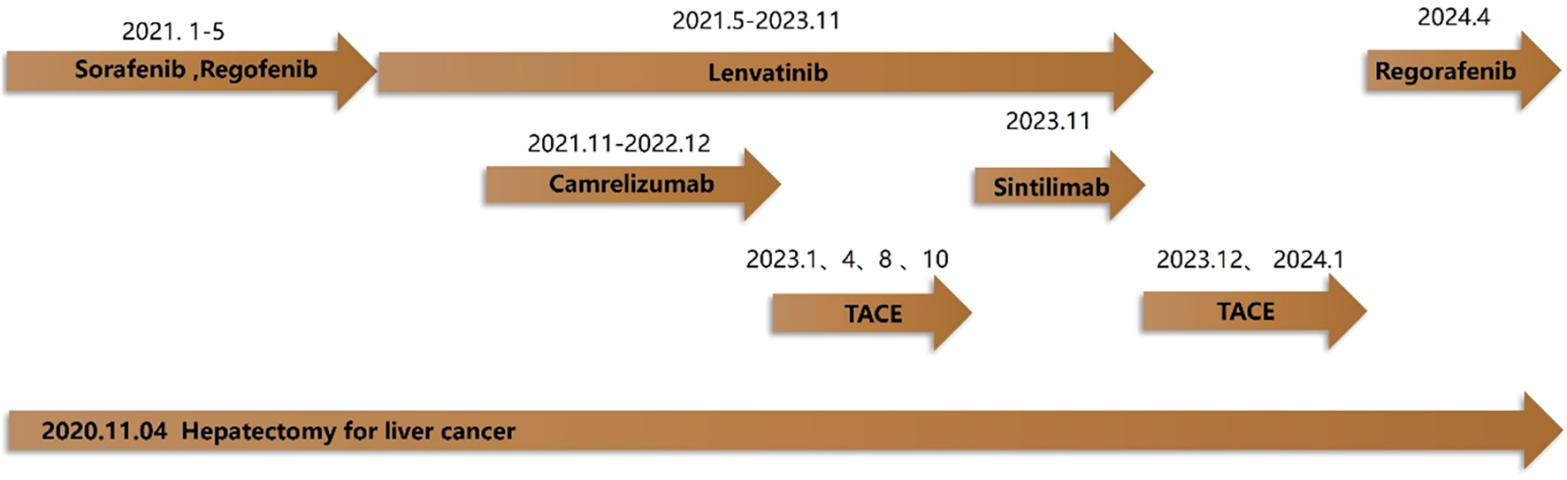
The HCC treatment process of the patient.

A middle - aged Han Chinese male, employed as a civil servant, was admitted for re - evaluation following over 3 years of comprehensive treatment after liver cancer resection, more than 2 years of targeted and immunotherapy for liver cancer, and over 8 months since the 6th transcatheter arterial chemoembolization (TACE) procedure (**Figure 1**).

Three years ago, the patient was diagnosed with HCC and underwent laparoscopic hepatectomy under general anesthesia. Starting from 2021, the patient received multiple targeted agents, including Sorafenib, Regorafenib, and Lenvatinib. Immunotherapy drugs, such as Camrelizumab and Sintilimab, were added in combination as the alpha - fetoprotein (AFP) levels fluctuated. Between 2023 and 2024, the patient underwent 6 TACE procedures. After the last discharge, the patient continued with Regorafenib for targeted maintenance therapy. On April 27, 2024, the patient suddenly lapsed into a coma due to severe hyponatremia (blood sodium level of 107.6 mmol/L). After appropriate treatment, the patient’s consciousness was restored, and intractable hyponatremia was reported. Subsequently, targeted and immunotherapy were suspended until the current admission. In the recent period, the patient’s diet, sleep, and body weight remained normal.

### 1.2 Past medical history

The patient has a 1-year history of Hashimoto’s thyroiditis, which has been treated with Euthyrox for a long period of time; he has also suffered from hepatitis B for more than 10 years, and is currently on antiviral therapy with tenofovir disoproxil fumarate, with a well-controlled viral load.

### 1.3 Physical examination upon admission

At the time of admission, the patient’s temperature was 36 °C, pulse was 70 beats/min, respiratory rate was 19 beats/min, and blood pressure was 110/73 mmHg. The patient was clear and mentally available, and he was cooperative in body checking and answering questions. The skin and mucous membranes were mildly yellowish, surgical scars were visible on the abdomen, and there were no liver palms or spider nevi. Cardiopulmonary examination did not show any abnormality, respiratory sounds were clear, no murmurs or arrhythmia were heard. Abdomen was distended, with mild tenderness, positive mobile turbidities, and no palpable masses. There was no edema in both lower limbs.

### 1.4 Admission diagnosis and treatment

The admission diagnoses included malignant liver tumor (stage IIb), decompensated hepatitis B cirrhosis (with splenomegaly and ascites), bile duct dilation, and Hashimoto’s thyroiditis. Initial treatment involved antiviral therapy with tenofovir, liver - protecting agents such as glutathione and ademetionine butanedisulfonate, diuretics including furosemide and spironolactone, as well as levothyroxine sodium tablets (“Euthyrox”) and caffeic acid tablets. After diagnostic abdominal paracentesis, piperacillin - tazobactam 4.5 g q8h was administered for anti - infection, plasma was used to correct coagulation, and tolvaptan was employed to address hyponatremia. Consultation with the endocrinology department led to the diagnosis of adrenal cortical insufficiency, hypothyroidism, and hyponatremia.

### 1.5 Admission examination results

The auxiliary examinations of the admitted patients are shown in **Table 1**. It should be particularly noted that the serum cortisol level in the morning (8:00 a.m.) is 5.16 nmol/L (reference range: 166–507 nmol/L), and the corresponding adrenocorticotropic hormone (ACTH) level is <1.50 pg/mL (reference range:7.2-63.3 pg/mL). The serum cortisol (COR) level of the previous patient on the morning of April 28th (8:00 a.m.) was 117 nmol/L (reference range:166–507 pg/mL), and the corresponding ACTH level was 14.3 pg/mL. This indicates that the patient developed adrenocortical insufficiency 4 months ago.

**Table 1:** Auxiliary examinations of patients on admission.

Virus and biochemical tests		Liver function and biochemical indicators		Ascites routine	
Hepatitis B virus DNA	<20.00 IU/ mL	AST	82↑ U/L	Ascites Color	Yellow
Blood ammonia	30.00 μmol/L	ALT	37 U/L	Character	Turbid
Abnormal prothrombin - II	10.00 mAU/ mL	GGT	115↑ U/L	Red Blood Cell Count	9000.00×10^6^/L
Alpha-fetoprotein	70.60↑ ng/ mL	ALP	207↑ U/L	Nucleated Cell Count	426×10^6^/L
Prothrombin time	18.40 seconds↑	Total bile acid	76.70↑ μmol/L	Percentage of Mononuclear Cells	93.00%
Prothrombin time activity	46.00%↓	Albumin	32.6↓ g/L	Percentage of Polymorphonuclear Cells	7.00%
Prothrombin standardized ratio	1.65 INR↑	Globulin	32.3 g/L	Rivalta Test	Negative
Indices related to the endocrine system		Total bilirubin	47.03↑ μmol/L	Ascites Biochemistry	
Cortisol	5.16 nmol/ L	Direct bilirubin	15.58↑ μmol/L	Adenosine Deaminase	5.81 U/L
Adrenocorticotropic Hormone	<1.50↓pg/ mL	Indirect bilirubin	31.45↑ μmol/L	Glucose	7.83 mmol/L
Free Triiodothyronine (FT3)	3.85 pmol/ L	Glucose	4.85 mmol/L	Chloride	110.1 mmol/L
Free Thyroxine (FT4)	8.38 pmol/ L	Creatinine	62.70 μmol/L	Protein Quantitation	11.80 g/L
Thyroid Stimulating Hormone (TSH)	8.6849 μIU/m L	eGFR	109.69	Blood Routine Tests	
Anti - Thyroglobulin Antibody	140	Uric acid	387 μmol/L	White blood cell count	2.48↓×10^9^/L
Anti - Thyroid Peroxidase Antibody	107	Calcium	2.18↓ mmol/L	Hemoglobin level	106.00↓ g/L
Thyrotropin Receptor Antibody	<0.80	Phosphorus	1.42 mmol/L	Platelet count	44.00↓×10^9^/L
		Magnesium	0.76 mmol/L	Percentage of neutrophils	51.60%
		Potassium	4.12 mmol/L	Percentage of eosinophils	4.40%
		Sodium	133.1↓ mmol/L	Absolute value of lymphocytes	0.91↓×10^9^/L
		Chloride	103.6 mmol/L	Absolute value of monocytes	0.17×10^9^/L
		Carbon dioxide	235 mmol/L	Absolute value of neutrophils	1.28↓×10^9^/L
		Osmotic pressure	265.92↓ mOsm/L	Absolute value of eosinophils	0.11×10^9^/L
		Anion gap	10.12↓ mmol/L		

## 2 Disease progression

Approximately 30 minutes after the start of piperacillin - tazobactam infusion, the patient developed an abrupt onset of fever, with the body temperature rising rapidly to 39°C, accompanied by severe chills that caused the entire body to shiver. Intense nausea and continuous retching were also present, although no vomiting occurred at that time. Suspecting a drug - related adverse reaction, the medical team promptly suspended Piperacillin - tazobactam and switched to Levofloxacin for anti - infection.

After one hour, the patient’s chills gradually subsided, and the body temperature decreased to 38°C. The nausea and retching also improved. However, laboratory tests revealed abnormal results in **Table 2**. The procalcitonin (PCT) were elevated to 0.17 ng/ml (reference range: 0-0.05 ng/mL), suggesting a possible inflammatory response. Interleukin-6 (IL-6) was significantly elevated to 1227.00 pg/ml (reference range: 0–7 pg/mL), suggesting that an immune-mediated process was underway. Blood glucose level decreased to 2.79 mmol/L (reference range: 3.9-6.1 mmol/L) and sodium level was 130.9 mmol/L (reference range: 137–147 mmol/L), which were both below the normal range. Although routine blood tests showed no significant changes in white blood cell (WBC) 3.9*10^9^/L (reference range: 3.5-9.5*10^9^/L), neutrophil percentage 58.7% (reference range: 40-75%), and C-reactive protein (CRP) 4.06 mg/L (reference range: 0–8 mg/L), these abnormal biochemical indicators were still cause for concern.

**Table 2:** Auxiliary examinations during disease progression.

The changing indicators (17:40 on August 16, 2024)		Blood gas analysis (03:41 on August 17, 2024)		Others (03:41 on August 17, 2024)	
Procalcitonin	0.17ng/mL↑	pH	7.41	High - sensitivity Troponin T	24.20↑ pg/mL
Interleukin - 6	1227.00pg/m L↑	Partial Pressure of Carbon Dioxide	34.00↓ mmHg	Creatine Kinase - MB	0.62 ng/mL
Glucose	2.79mmol/L↓	Partial Pressure of Oxygen	98.00 mmHg	Myoglobin	81.30↑ ng/mL
Sodium	130.9mmol/L↓	Potassium Ion	3.80 mmol/L	Procalcitonin	20.30↑ ng/mL
Blood Routine Tests(03:41 on August 17, 2024)		Sodium Ion	128.00↓ mmol/L	Interleukin - 6	1621.00↑ pg/mL
WBC	3.27↓ 10^9^/L	Chloride Ion	103.00 mmol/L	N-pro-BNP	966.00↑ pg/mL
RBC	316↓ 10¹²/L	Ionized Calcium	1.08↓ mmol/L	Alanine Aminotransferase	30 U/L
Hb	101↓ g/L	Glucose	7.00↑ mmol/L	Gamma - Glutamyl Transpeptidase	77↑ U/L
PLT	37↓ 10^9^/L	Lactate	2.10↑ mmol/L	Total Protein	48.5↓ g/L
Percentage of Lymphocytes	12.6↓ %	Hemoglobin	104.00↓ g/L	Albumin	24.0↓ g/L
Percentage of Monocytes	6.80%	Oxyhemoglobin	95.40%	Globulin	24.5 g/L
Percentage of Neutrophils	78.4↑ %	Carboxyhemoglobin	2.60↑ %	Ratio of Albumin to Globulin	0.98↓
Percentage of Eosinophils	2.20%	Reduced Hemoglobin	1.00%	Total Bilirubin	43.55↑ μmol/L
Percentage of Basophils	0.00%	Methemoglobin	1.10%	Direct Bilirubin	18.37↑ μmol/L
Absolute Value of Lymphocytes	0.41↓ 10^9^/L	Oxygen Saturation	99.00↑	Indirect Bilirubin	25.18↑ μmol/L
Absolute Value of Monocytes	0.22 10^9^/L	Total Carbon Dioxide	22.60↓ mmol/L	Glucose	6.88↑ mmol/L
Absolute Value of Neutrophils	2.56 10^9^/L	Base Excess in Extracellular Fluid	-3.00 mmol/L	Creatinine	131.20↑ μmol/L
Absolute Value of Eosinophils	0.07 10^9^/L	Base Excess in Whole Blood	-2.50 mmol/L	Uric Acid	361 μmol/L
		Anion Gap	7.00↓ mmol/L	Cystatin	253↑ mg/L
		Alveolar Oxygen Pressure	164.00 mmHg	Beta2 - Microglobulin	7.70↑ mg/L
		Bicarbonate Concentration	21.60 mmol/L	β - Hydroxybutyric Acid	0.03 mmol/L
		Standard Bicarbonate	23.00 mmol/L	Retinol - binding Protein	5.90↓ mg/L
		Respiratory Index	0.7	Complement C1q	96.50↓ mg/L
		Hematocrit	31.00↓ %	Calcium	1.96↓ mmol/L
		Oxygen Inhalation Concentration	29	Phosphorus	0.69↓ mmol/L
		Body Temperature	37.10°C		
		Atmospheric Pressure	760 mmHg		
		Difference between Alveolar and Arterial Oxygen Pressure	66.00 mmHg		
		Ratio of Arterial Oxygen Pressure to Alveolar Oxygen Pressure	0.6		

Later in the evening, at 21:00, the patient’s condition deteriorated again. The patient had three episodes of watery diarrhea, each of small volume. Subsequently, the patient vomited twice, with the vomitus consisting of gastric contents and previously ingested oral medications. The body temperature remained at 38°C, without chills, but with minimal sweating. Physical examination at this time showed that the patient was still conscious but in a relatively weakened mental state. The blood pressure was 110/70 mmHg, the heart rate was 65 beats per minute, the pulse oxygen saturation was 96%, and the respiratory rate was 20 breaths per minute. To address the deteriorating condition, the medical team provided oxygen inhalation and initiated fluid replacement therapy while closely monitoring the blood pressure.

On August 17, 2024, at 1:00 a.m., the patient experienced a more critical situation. The patient suddenly developed acute abdominal pain with severe pain in the lower back and limbs. The patient clearly stated that the pain was not metastatic right lower abdominal pain, and there were no symptoms of shortness of breath, chest tightness, cough, or dyspnea. Body temperature remained at 39 °C. Physical examination showed that the patient was alert but in severe pain, pale and with a distressed expression. The skin was slightly moist with no blotches or petechiae. Breath sounds were diminished in both lungs, with no obvious dry or wet rales. Bowel sounds were normal, but there was marked percussion pain in the renal region. The abdomen was rigid with marked periumbilical tenderness and rebound pain. McBurney’s point was not indurated and shift retardation remained positive. There was no edema of the lower extremities. Cardiac monitoring showed a marked drop in blood pressure to 70/42 mmHg, a heart rate of 79 beats per minute, a pulse oximetry of 95%, and a respiratory rate of 18 breaths per minute. The electrocardiogram showed normal sinus rhythm with no significant arrhythmias and consistent blood pressure readings on both sides. Despite fluid resuscitation with crystalloid and colloid fluids, the patient’s blood pressure remained low, prompting the medical team to urgently contact a multidisciplinary consultation, including endocrinology, intensive care medicine, and gastroenterology.

**Figure 2:**
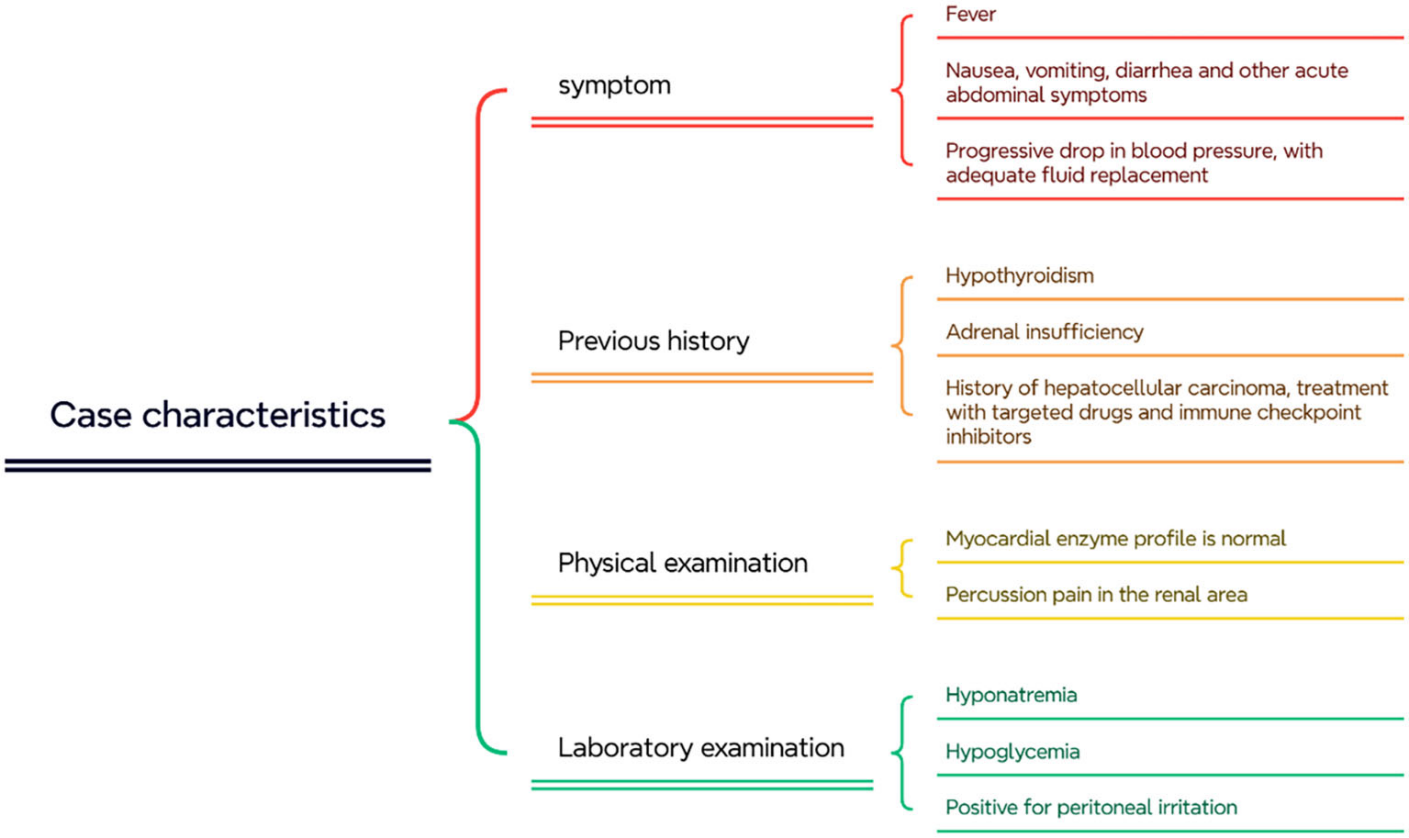
Summary of the characteristics of the patient's disease progression.

After a thorough review of the patient’s case features (**Figure 2**) and in-depth multidisciplinary discussion, we zeroed in on two primary diagnoses: 1. adrenal crisis (the patient had adrenal insufficiency and had been on ICIs); and 2. septic shock (the patient had a well-defined infection, primarily peritonitis). Based on the laboratory tests present at the time (shown in **Table 2**), our detailed differential between sepsis and adrenal crisis (shown in **Table 3**) still did not rule out either diagnosis. We did more detailed laboratory tests as shown in **Table 2**. It is worth mentioning that we sent blood bacterial cultures for testing multiple times but did not obtain positive results. Therefore, looking back on the entire treatment process, we further confirmed the diagnosis of adrenal crisis.

**Table 3:** Differential points between adrenal crisis caused by ICIs and septic shock.

Main differential dimensions	Specific differential points	Adrenal crisis caused by ICIs	Septic shock
Basic Information	Definition	A critical condition resulting from adrenal cortical hypofunction triggered by the use of immune checkpoint inhibitors (ICIs)	A shock state caused by severe infection leading to systemic inflammatory response syndrome
Etiology	Pathogenic Factors	Immune - mediated adrenal injury after the use of ICIs, resulting in adrenal cortical hypofunction	Severe infection caused by common pathogens (Gram - negative bacteria, Gram - positive bacteria, fungi, etc.)
Medical History Characteristics	Key Points of Inquiry	There is a clear history of ICI use. Pay attention to the duration, dosage of ICI use, and whether it is combined with other drugs. Symptoms usually gradually appear after the use of ICIs for a period of time	There is a clear history of exposure to infectious foci recently. The condition deteriorates rapidly after the appearance of infectious symptoms and progresses to shock within a short period (hours to days)
Clinical Manifestations	Fever Condition	Mostly low - grade or moderate - grade fever	Often high fever, and the body temperature may not rise in some elderly, frail, or immunocompromised patients
	Circulatory System Manifestations	Hypotension, not obvious heart rate increase, and poor response to fluid replacement and vasoactive drugs	Warm shock in the early stage (warm and dry skin, normal or low - normal blood pressure, increased heart rate), and progresses to cold shock in the later stage (pale and cold - wet skin, decreased blood pressure, significantly increased heart rate, thready and rapid pulse)
	Digestive System Manifestations	Severe nausea, vomiting, abdominal pain, and diarrhea, often accompanied by dehydration and electrolyte disorders	Nausea, vomiting, and abdominal pain are relatively mild
	Nervous System Manifestations	Listlessness, drowsiness, coma, etc., may be accompanied by headache and restlessness	Restlessness and delirium in the early stage, and consciousness disorders in the later stage, but generally less severe than in adrenal crisis
	Other Characteristics	May present with skin hyperpigmentation (exposed areas, areola, scars, etc.); some patients may have systemic symptoms such as fatigue and muscle - joint pain	Manifestations related to the primary infectious focus, such as cough, expectoration (pulmonary infection), frequent urination, urgency, and pain during urination (urinary tract infection), etc.
Auxiliary Examinations	Imaging Examinations	Adrenal CT or MRI may show adrenal enlargement or density changes (due to immune injury)	Chest X - ray or CT, abdominal ultrasound, etc. are helpful for finding the primary infectious focus, such as pulmonary inflammation, abdominal abscess, etc.; adrenal imaging generally has no specific changes (unless the infection directly involves the adrenal gland)
	Routine Laboratory Examinations	White blood cell count is normal or slightly increased, and the proportion of neutrophils is normal or slightly higher; there are hyponatremia, hyperkalemia, and hypoglycemia; coagulation function is generally not significantly abnormal; blood cortisol level is decreased, and adrenocorticotropic hormone level is increased	White blood cell count is significantly increased, the proportion of neutrophils is increased, there is a left - shift of the nucleus, and toxic granules can be seen; metabolic acidosis, liver and kidney function impairment occur, and electrolyte disorders are relatively mild; coagulation dysfunction may occur; there are generally no obvious hormonal level abnormalities, and stress - induced cortisol increase may occur in severe infections
	Special Laboratory Examinations	In the CRH stimulation test, there is no significant increase in blood ACTH and cortisol levels after the administration of CRH; cytokine levels (such as TNF - α, IL - 6, etc.) generally do not increase significantly (when not combined with infection); PCT generally does not increase (when not combined with infection)	In the CRH stimulation test, ACTH and cortisol levels usually show a normal stress - induced increase (when there are no other endocrine abnormalities); the levels of inflammatory cytokines in the body (such as TNF - α, IL - 6, etc.) are significantly increased; PCT is significantly increased and is related to the severity of the infection
Treatment Principles	Treatment Direction	Immediately discontinue ICIs, supplement glucocorticoids (such as hydrocortisone), supplement normal saline, glucose, and correct water and electrolyte disorders and hypoglycemia	Anti - infection (using effective antibiotics), anti - shock (supplementing blood volume, correcting acidosis, applying vasoactive drugs, etc.)
Treatment Response	Observation Points	After discontinuing ICIs and supplementing glucocorticoids, the symptoms improve rapidly, such as blood pressure rising and mental state improving, suggesting that this disease may be present	After active anti - infection and anti - shock treatment, the condition gradually stabilizes, which is more likely to be this disease; experimental treatment needs to be carefully evaluated and closely monitored to avoid delaying the condition

**Figure 3:**
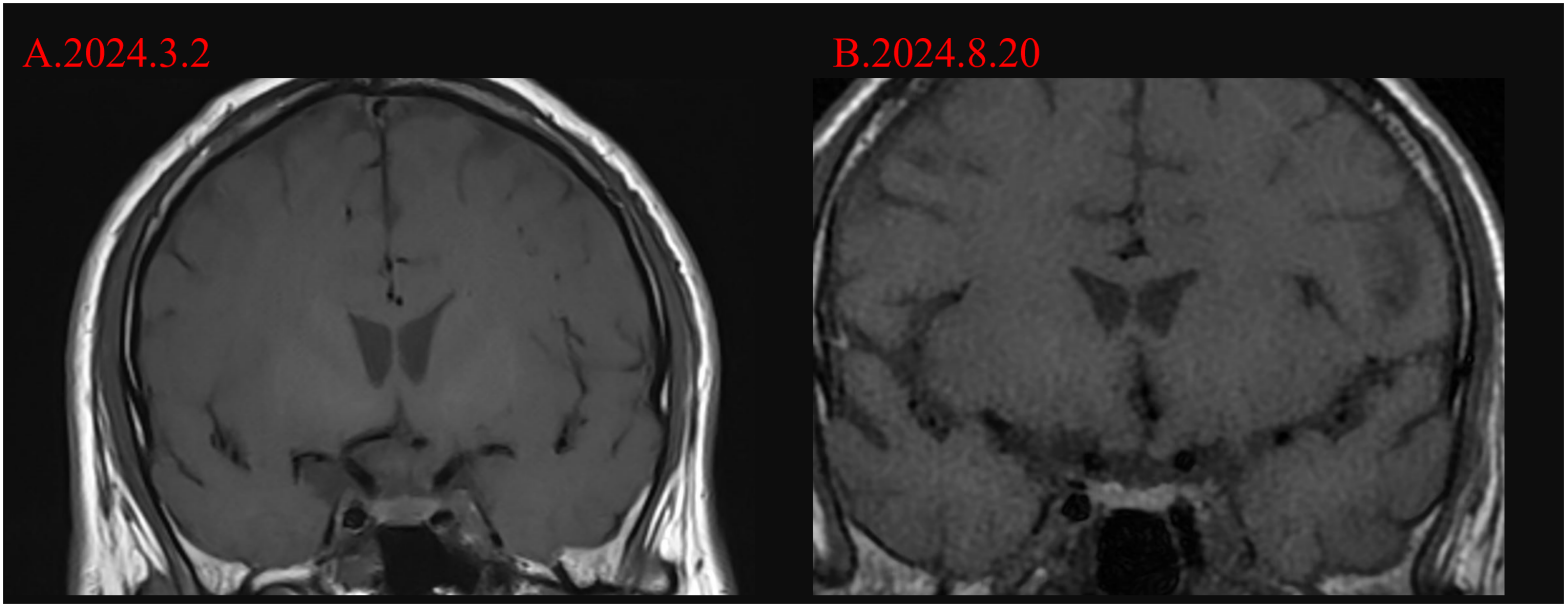
MRI changes of the pituitary gland before and after the occurrence of adrenal crisis in the patient. **(A)** shows the T2-weighted image of the pituitary gland of the patient before the occurrence of adrenal crisis (March 2, 2024), at this time the patient did not show any manifestations of adrenal cortical insufficiency such as hyponatremia; **(B)** shows the T2-weighted image of the pituitary gland of the patient on the third day after the occurrence of adrenal crisis (August 20, 2024), at this time the pituitary gland was only slightly thinner, and no obvious abnormalities were observed.

**Figure 4:**
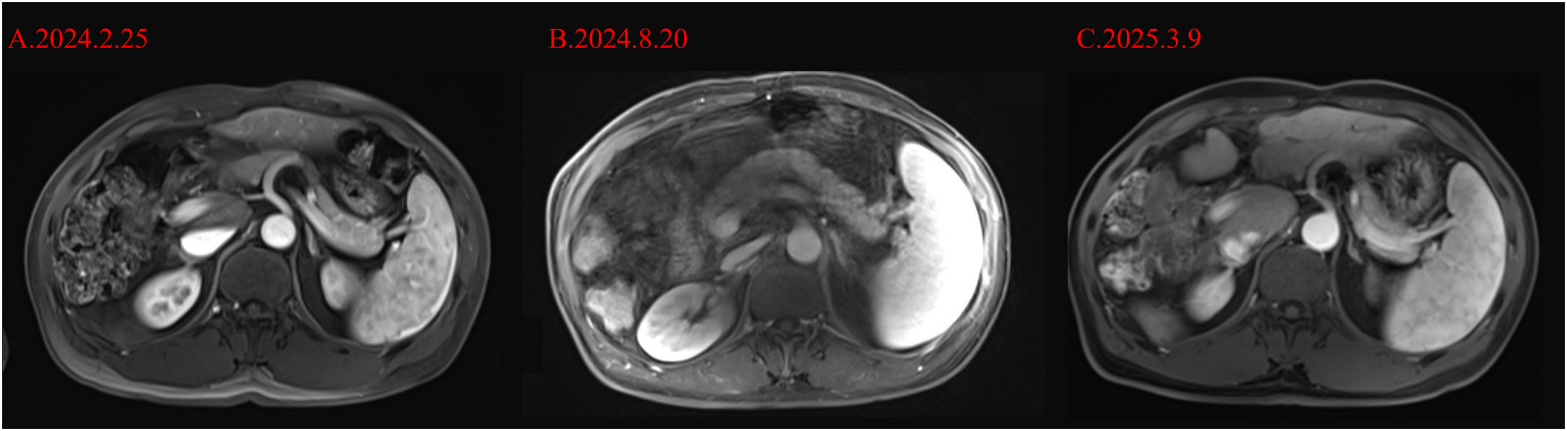
MRI changes of the adrenal glands before and after the occurrence of adrenal crisis in the patient. **(A)** shows the MRI image of the adrenal glands of the patient before the occurrence of adrenal crisis (2024.2.25), **(B)** shows the MRI image of the pituitary gland of the patient on the third day after the occurrence of adrenal crisis (2024.8.20), and **(C)** shows the MRI image of the adrenal glands of the patient about half a year after the occurrence of adrenal crisis.

Once adrenal crisis was taken into account, we immediately administered 100 mg of hydrocortisone, adequate fluid resuscitation, and vasopressor medication to maintain blood pressure in accordance with the treatment guidelines. The patient’s blood pressure fluctuated at 80-90/55–65 mmHg during the night. In addition, due to definite peritonitis in the patient, there were markedly elevated PCT (20.3 ng/mL) and IL-6 (1621.00 pg/mL), as well as decreased WBC (3.27* 10^9^/L), a neutrophil percentage of 78.4%, and a CRP of 8.52 mg/L, meropenem 1 g q8h was still administered for anti-infective. After confirming the supplementation of hydrocortisone, the COR value we remeasured the next day was 1261 nmol/L and ACTH was less than 1.5 pg/ml. We continued to administer 200 mg of hydrocortisone for 24 hours for anti-infection and antihypertensive treatment. On August 18, 2024, the patient’s blood pressure stabilized at 120/71 mmHg, and we discontinued dopamine. On August 19, 2024, the patient’s blood pressure was stable. Due to ascites, a diuretic was added. Hydrocortisone was reduced to 100 mg for intravenous infusion every day and anti-infection treatment was administered. We conducted abdominal and adrenal magnetic resonance imaging (MRI) as well as pituitary magnetic resonance imaging for the cause. No obvious abnormalities were found in the result (**Figures 3**, **4**). Three days later, it was changed to oral hydrocortisone acetate tablets 40 mg bid. At the same time, the antibiotics were reduced to oral levofloxacin tablets 0.5 g qd, levothyroxine sodium tablets 100 μg qd, ursodeoxycholic acid tablets 250 mg tid and concentrated sodium treatment were continued. On August 28, 2024, the COR was reexamined at 338 nmol/L and the ACTH was less than 1.5 pg/ml. After hormone supplementation, the patient no longer experienced symptoms.

## 3 Follow - up and outcome

We conducted a six-month follow-up, during which the patient had a good appetite, improved fatigue, and a stable physical condition. Relevant laboratory and clinical indicators were stable, such as COR 102 nmol/L, blood glucose 4.9 mmol/L, and blood sodium 137.1 mmol/L (March 9, 2025), which indicated that the patient’s complex condition was under positive control. The patient continued to take hydrocortisone acetate tablets, levothyroxine tablets, and polyene phosphatidylcholine, and discontinued treatment with targeted drugs and ICIs.

## 4 Discussion

This case involves a patient with liver cancer who had a history of “hepatitis B” and “Hashimoto’s thyroiditis”, as well as a period of hyponatremia. In addition, the patient had a history of immune checkpoint inhibitor treatment and experienced multiple ICI - related liver injuries. The patient’s general condition was fair, but examinations indicated the presence of peritonitis and adrenocortical insufficiency, with no prior history of steroid (glucocorticoid) use. The next day after admission, the patient developed a fever. After ruling out adverse reactions caused by antibacterial drugs, the patient subsequently experienced persistent fever, loss of appetite, nausea, vomiting, abdominal pain, diarrhea, listlessness, tachycardia, hypotension, and shock. These symptoms are not specific. Especially considering the patient had decompensated liver cirrhosis complicated by intra - abdominal infection, similar symptoms could also occur when the disease progresses to septic shock. However, laboratory tests showed a decrease in plasma cortisol levels, along with electrolyte abnormalities such as hypoglycemia and hyponatremia, which caught our attention. After immediately administering hydrocortisone, the patient’s vital signs gradually stabilized. At the time of the patient’s fever we took blood cultures and ascites cultures, both of which were negative. Therefore, we diagnosed this series of changes in this patient as adrenal crisis. This resuscitation process was also very challenging for the hepatologists.

Although the patient underwent surgery, TACE, and small molecule targeted drugs, but because the targeted drugs are multi-target tyrosine kinase inhibitors, which mainly play an anti-tumor effect by inhibiting various enzymes related to tumor growth and angiogenesis, according to our investigation, there is insufficient evidence that these drugs and treatments are related to adrenal insufficiency (4, 5). However, the incidence of adverse events associated with the use of immune checkpoint inhibitors is on the rise. Randomized controlled trials have demonstrated an incidence of any IRAE in 54–96% of those treated with ICI therapy (6). In a comprehensive meta-analysis that did not incorporate any hepatocellular carcinoma (HCC) trials, the most commonly affected target organs by immune-related adverse events (irAEs) during cytotoxic T-lymphocyte-associated antigen 4 (CTLA-4) inhibition were the skin (44%) and the gastrointestinal tract (35%) (7). The endocrine glands and the liver were affected in 6% and 5% of cases, respectively. In another meta-analysis encompassing nearly 3,000 treated patients, the most frequently targeted organs by irAEs during programmed death-1 (PD-1) or programmed death-ligand 1 (PD-L1) inhibition were the skin (pruritus 10%, rash 11%), the gastrointestinal tract (diarrhea 11%), and the thyroid (hypothyroidism 7%) (8). Endocrine disorders include hypothyroidism, hyperthyroidism, pituitary inflammation, primary adrenal insufficiency (PAI), and insulin-deficient diabetes (9). Sintilimab (10) and Camrelizumab (11) and TA regimen (atilizumab combined with bevacizumab) (12) are used as first-line drugs for the treatment of hepatocellular carcinoma, and they are humanized anti-programmed death receptor 1 (PD-1) monoclonal antibodies. They mainly work by blocking the binding of PD - 1 to its ligand PD - L1, relieving the inhibitory effect of the PD - 1 pathway on T cells, thereby activating T - cell functions and enhancing the immune system’s attack on tumor cells (13–15). We searched for case reports of adrenal crisis due to tislelizumab applied to other tumors in **Table 4** (16–18), like lung squamous cell carcinoma and bladder cancer patients. It mostly occurs during the middle to late stages of treatment (median 7 cycles) or 5 months after discontinuation of treatment. All cases require permanent discontinuation of immunotherapy, and 5/6 patients suffer irreversible damage to adrenal function, requiring long-term hormone replacement.

**Table 4:** Case information.

Case source	Age/sex	Cancer type	ICI regimen	Onset time	Main clinical manifestations	Key diagnostic indicators	Treatment	Outcome
Wang et al. (2024)(17)	51M	Right lung squamous cell carcinoma (IVA, cT4N3M1a)	Tislelizumab + Albumin-bound paclitaxel/Carboplatin (Chemo combo)	Cycle 4	Non-specific symptoms	↓Cortisol, ↓ACTH; ↑TSH, ↑FSH, ↑PRL, ↑GH, ↓IGF-1; MRI negative (no hypophysitis)	Prednisone acetate + Levothyroxine	>8 months replacement therapy, cortisol unrecovered, ICI not restarted
	74M	Left lung squamous cell carcinoma (IVA, cT4N0M1b, rib metastasis)	Tislelizumab monotherapy	Cycle 9	Non-specific symptoms	↓Cortisol, ↓ACTH; Imaging not performed	Prednisone acetate	>7 months replacement therapy, cortisol unrecovered, ICI not restarted
	56M	Right lung squamous cell carcinoma (IIIA, pT2aN2M0)	Neoadjuvant (Tislelizumab + Albumin-bound paclitaxel/Carboplatin) → Adjuvant Tislelizumab	Cycle 8 (maintenance phase)	Fatigue, vomiting, weight loss (2.5kg in 1 month)	↓Cortisol, ↓ACTH; Imaging not performed	Hydrocortisone	Temporary recovery after replacement, cortisol ↓ again after ICI restart, ICI permanently discontinued
Zhang et al. (2023)15	72M	Bladder cancer (T1N0M0) + Prostate cancer (T2N0M0)	Tislelizumab monotherapy (200mg q3w, 5 doses) + Intravesical gemcitabine	5 months after discontinuation	Progressive nausea/vomiting, fatigue, anorexia, weight loss (19kg in 4 months)	↓ACTH (<3.0 pg/mL), ↓Cortisol (<0.5 μg/dL); ↑TSH (140.48 μIU/mL), ↓FT4 (0.13 ng/dL); ↓Testosterone (1.64 ng/mL), low-normal LH/FSH; PET/CT: Thickened pituitary stalk ↑uptake, MRI: Enlarged heterogeneous pituitary	IV Hydrocortisone 50mg/day → Oral 40mg/day + Levothyroxine 12.5μg/day	After 2mo: Cortisol 7.8μg/dL, TSH still ↑(104.87 μIU/mL), Weight ↑5kg
Wei et al. (2024)17	58M	Bladder urothelial carcinoma (low-grade non-invasive)	Tislelizumab (200mg q3w)	Cycle 8 (7 months)	Recurrent syncope, hyperpyrexia, hypotension (50-60mmHg), confusion	↓Cortisol (0.16-0.15 μg/dl), ↓ACTH (4.47-2.16 pg/ml); Pituitary MRI normal	Acute: Hydrocortisone 0.15g IV bid + fluid resuscitation; Maintenance: Prednisone 10mg am + 5mg pm	Symptoms resolved at 3mo, adrenal function unrecovered, ICI stopped, switched to ADC

Adrenal crisis is a relatively rare and serious immune checkpoint inhibitor (ICI)-related adverse event that usually occurs in patients with adrenocortical insufficiency. According to the Common Terminology Criteria for Adverse Events (CTCAE), adrenal cortical insufficiency is usually a grade 1–2 immune - related adverse event (irAE), while adrenal crisis is a grade 3–4 irAE (19). A large meta - analysis of 160 clinical trials involving 40,432 patients reported that among patients using ICIs, the estimated incidence rates of adrenal cortical insufficiency and hypophysitis were 2.43% and 3.25% respectively. Using a random - effects model, the incidence rates of all - grade and severe - grade adrenal cortical hypofunction were 2.43% (95% CI, 1.73% - 3.22%) and 0.15% (95% CI, 0.05% - 0.29%) (20). Adrenal cortical insufficiency usually presents with some non - specific clinical symptoms, including fatigue, anorexia, nausea, abdominal pain, and diarrhea (21). These symptoms are difficult to distinguish from the complications of liver cancer patients. The symptoms of adrenal crisis are more severe. Based on the above - mentioned symptoms, there may be listlessness, tachycardia, hypotension, shock, and even coma. These symptoms are difficult to distinguish from those of septic shock. Therefore, laboratory tests are essential for the diagnosis of adrenal crisis, and attention should be paid to the examination of cortisol and electrolytes.

Immune checkpoint inhibitor-induced adrenal insufficiency (AI) can be divided into primary AI (adrenocortical destruction) and secondary AI (ACTH deficiency due to pituitary failure) according to the site of the lesion, and the mechanisms and characteristics of the two are significantly different. Secondary AI is the most common type of ICI-associated AI, mainly caused by ICI-induced pituitary inflammation, and its pathogenesis is closely related to autoimmune response (22), especially seen with CTLA-4 inhibitors (e.g., ipilimumab) and combination therapies (CTLA-4 + PD-1/PD-L1 inhibitors). Preclinical and clinical evidence supports this mechanism (23, 24): anti-CTLA-4 monoclonal antibody induces pituitary inflammation through complement-mediated cytotoxicity in a mouse model; autopsy studies have shown strong CTLA-4 expression in the pituitary gland of the patients as well as pathological features consistent with both type II (antibody-mediated) and type IV (T-cell-mediated) hypersensitivity; and antipituitary hormone antibodies (e.g., anti-ACTH antibodies) are detected in the patient serum. Pituitary inflammation leads to insufficient ACTH secretion (incidence up to 75%), which in turn causes atrophy of the adrenocortical zona fasciculata and decreased cortisol synthesis, manifesting as hypocortisolism with low/normal ACTH, often coexisting with other anterior pituitary hormone deficiencies; recovery of the ACTH axis function is rare, and long-term glucocorticoid replacement is usually required.

In contrast, primary AI is relatively rare (~0.7% overall, up to 4.2% with combination therapy), and the mechanism is that ICI (especially with combination therapy) triggers autoimmune adrenal inflammation that directly destroys the adrenal cortex (25). Due to the low incidence of PAI associated with ICPis, the number of cases is small, the follow-up is short, and the characteristics of the population and the risk factors have not yet been clarified. Known risk factors include a history of comorbid autoimmune disease, use of CTLA-4 inhibitors, and chronic kidney disease stage 3 or higher. Drug dosage may also influence morbidity, with studies showing a higher risk of PAI at doses of ibritumomab above 5 mg/kg (26).This is similar to traditional Addison’s disease, resulting from adrenocortical destruction mediated by T cells, etc. ICI may break tolerance to adrenal autoantigens (e.g.,21-hydroxylase), involving susceptibility genes (e.g. CTLA-4, PDCD1) and specific HLA haplotypes (e.g., HLA-DR3-DQ2) (27, 28). Primary AI results in a severe deficiency in both cortisol and saline corticosteroid (aldosterone) secretion, which manifests as low cortisol with high ACTH, and characteristic symptoms include malaise, hypotension, nausea and vomiting, hyponatremia, hyperkalemia, and hyperpigmentation of the skin and mucous membranes. Adrenocortical destruction is usually irreversible and requires lifelong combined replacement of physiologic doses of glucocorticoids and saline corticosteroids (fludrocortisone) and strict patient education in stress management. The key points of differentiation between the two are: secondary AI originating from pituitary inflammation (lesion in the pituitary gland, ACTH deficiency), with biochemical hallmarks of low cortisol + low/normal ACTH, electrolyte disturbances mainly hyponatremia, no hyperpigmentation, usually preserved saline corticosteroid function, and rare restoration of the ACTH axis; and primary AI originating from adrenal inflammation (lesion in the adrenal gland), with biochemical hallmarks of low cortisol + high ACTH, characterized by low sodium and high potassium and hyperpigmentation, with salicorticoid deficiency requiring replacement, largely irreversible (25).

The American Society of Clinical Oncology (ASCO) guidelines (29) state that routine endocrine examinations should be performed on patients receiving ICI treatment to assess endocrine glands or organs, which is of great importance. We firstly found the diagnosis of adrenal cortical hypofunction through the examination of the patient’s cortisol and ACTH, which provided an effective clue for our diagnosis. Several studies have shown that a history of prior adrenal crisis is a predisposing factor for recurrent adrenal crisis in patients with adrenocortical insufficiency (31). In reviewing the patient’s medical history, we found that the patient had an episode of hyponatremia with impaired consciousness 5 months prior to admission, when the serum cortisol (8:00 a.m.) level was 117 nmol/L (reference range: 166–507 nmol/L), and the corresponding ACTH level was 14.3 pg/ml pg/mL (reference range: 7.2-63.3 pg/mL), which suggested that the patient already had adrenocortical insufficiency at that time, and what we could not confirm was whether the patient’s impaired consciousness was a neurologic manifestation of irAEs or due to hyponatremia. However, the emergency physician did not realize that this could be related to irAEs and did not supplement hydrocortisone in a timely manner, which may be another important reason for the development of this crisis and needs to be brought to the attention of emergency physicians as well as physicians in related specialties.

The timing of IRAEs varies, typically occurring within weeks to months of treatment commencement. The median time to onset of moderate to severe endocrine disorders treated with ipilimumab was 1.75–5 months, and that treated with PD-1 inhibitors was 1.4-4.9 months (30, 31). Moreover, adrenal dysfunction may occur at any time during or even after ICIs (32). Importantly, this irAE can occur several months after discontinuing an ICI (33). An increasing number of clinical cases have proven that endocrine diseases such as delayed - onset adrenal crisis (AC) can occur after the termination of ICI treatment, which also demonstrates that the anti - tumor effect of ICIs can be expressed in the body for a long time (34, 35). Therefore, even after the discontinuation of ICIs, it is recommended to always be vigilant about the possibility of irAEs.

In addition, in this patient, we found that apart from receiving 8 cycles of PD - 1 treatment, there were no other related causes and predisposing factors. Although the drug had been discontinued for 4 months, the damage seemed to be persistent. Patients with pre-existing autoimmune diseases have a higher risk of autoimmune disease deterioration and may also experience unrelated irAEs (36). The patient has an autoimmune disease basis for Hashimoto’s thyroiditis, which is a high risk factor.

Since the patient also had a decrease in thyroid hormone levels, we considered the possibility of immune - related hypophysitis (irH). It is defined as the occurrence of functional defects in one or more pituitary axes in patients receiving ICI treatment, with or without mild pituitary MRI abnormalities (37, 38).The diagnostic criteria proposed by Nguyen (39) et al. include central adrenal cortical insufficiency or central hypothyroidism, and MRI findings consistent with irH, or the presence of symptoms of central adrenal cortical insufficiency and hypothyroidism (such as headache or fatigue), but no abnormalities were found on MRI or MRI was not performed. We completed the detection of other hormone levels, with the results as follows: growth hormone (0’) 11.60 ng/ml (elevated), 17α-hydroxyprogesterone 0.48 ng/ml, follicle-stimulating hormone 8.23 mIU/ml, luteinizing hormone 13.19 mIU/ml (elevated), estradiol 38.69 pg/ml, progesterone 0.08 ng/ml, testosterone 4.29 ng/ml, prolactin 35.89 ng/ml (elevated), sex hormone-binding globulin 34.31 nmol/L, free testosterone index 43.39%, and anti-Müllerian hormone 2.460 ng/ml. None of the aforementioned indicators showed clinically significant abnormalities. Unfortunately, as of the submission of this manuscript, considering safety and the patient’s autonomous wishes, the ACTH stimulation test was not performed on the patient. Research shows that 77% of patients with IRH have pituitary enlargement, stalk thickening, and homogeneous or heterogeneous contrast enhancement on MRI, while 23% - 33% of patients show no abnormalities on MRI (24). We also conducted imaging examinations of the pituitary and adrenal glands, and no special findings were observed in this patient. Studies have found that compared with CTLA - 4 inhibitors, pituitary induced by PD - 1/PD - L1 inhibitors may lack typical pituitary enlargement and present a normal pituitary morphology (29, 39). We performed an MRI of the pituitary gland and adrenal glands in this patient, and the results were unremarkable as shown in **Figures 3**, **4**. In general, when endocrine examinations suggest central adrenal cortical hypofunction, hormone replacement therapy should not be delayed due to waiting for pituitary MRI results.

In terms of treatment, the key to treating adrenal crisis is to rapidly replenish glucocorticoids and correct electrolyte disorders (40). Usually, it is necessary to immediately inject hydrocortisone intravenously, and then continue infusion or give maintenance doses regularly. At the same time, it is necessary to actively replenish normal saline to correct dehydration and electrolyte imbalance. During the treatment process, it is also necessary to actively control predisposing factors such as infections and provide systemic supportive treatment. When an immune - therapy - related adrenal crisis occurs, in addition to supportive fluid therapy, an initial intravenous or intramuscular bolus injection of 100 mg of hydrocortisone is required, along with continuous intravenous infusion of 200 mg of hydrocortisone once every 24 hours (per day) or 50 mg of hydrocortisone every 6 hours (or 50 mg, four times a day). The recommended duration is 24–48 hours. Alternatively, after the initial bolus dose, a continuous infusion of 200 mg/24 h can be used until the patient can take hydrocortisone orally (3). It should be noted that dexamethasone is not a suitable substitute for glucocorticoids because it lacks mineralocorticoid activity. Our patient had no history of underlying endocrine diseases such as diabetes, so there were no specific restrictions on the dosage of cortisol. For diabetic patients, choosing an appropriate cortisol dosage to maximize benefits and reduce related side effects is a challenge. For adults, when the patient’s condition is stable, glucocorticoid replacement therapy should be the main treatment. Hydrocortisone can be selected and administered three times a day (or occasionally twice a day). For example, 10 mg can be taken immediately after waking up, 5 mg at noon, and 5 mg in the afternoon, or 15 mg after waking up and 5 mg at noon (40, 41). During subsequent follow - up, the dosage of hydrocortisone is gradually reduced according to the patient’s clinical manifestations, and blood pressure and recurrence of clinical symptoms are closely monitored. Both the rescue and treatment of this patient were conducted under endocrinologists’ guidance, with the overall treatment plan aligned with the core principles of standard guidelines. We recommend immediate consultation with endocrinologists for intervention and treatment guidance once adrenal crisis is suspected, followed by subsequent follow-up in the Department of Endocrinology. According to relevant guidelines or recommendations from NCBI, patients need to be on long-term hydrocortisone and monitored for COR. Additionally, patients will need to be educated on sick day rules and be provided with medical alert bracelets, and have high-dose corticosteroids for emergency purposes (9, 42).

There are several issues that require in-depth consideration. First, it has been suggested that the occurrence of irAEs may be a positive predictor of treatment response, and that there is a positive correlation between the occurrence of immune-related adverse events (irAEs) induced by immune checkpoint inhibitor (ICI) therapy and improved tumor response and survival rates (43). Theoretically, during the treatment with ICI, as the immune system’s tolerance to triggering irAEs decreases, its ability to recognize and kill cancer cells indeed increases. Before admission, we evaluated the patient’s liver cancer condition and found no metastatic or recurrent tumor lesions, indicating that the patient had a complete response to the immune checkpoint inhibitor treatment. We have followed up the patient for half a year after the occurrence of adrenal crisis (as of the time of publication) and have not found any signs of HCC recurrence. Therefore, it seems necessary to continue the treatment with immune checkpoint inhibitors. However, resuming the treatment with immune checkpoint inhibitors requires considering many factors. Currently, it is believed that after the disappearance of adverse reactions, it is necessary to comprehensively consider the previous tumor response, treatment duration, type and severity of toxicity, time of toxicity regression, availability of alternative therapies, the patient’s condition, and the patient’s acceptance before deciding whether to restart the trea. However, the recovery of the pituitary-adrenal axis is very rare (44, 45).

Second, current medical tests are unable to distinguish between immune-related and non-immune-related causes, and the lack of specific immunobiological markers makes it difficult for clinicians to detect irAEs (46). The management of ICI - related irAEs requires the collaboration of a multi - disciplinary team and timely assessment of the patient’s basic endocrine hormone levels. Before treatment with PD - 1/PD - L1 inhibitors, patients should be carefully questioned about their history of endocrine and autoimmune diseases, undergo reasonable baseline screening, regularly monitor changes in endocrine indicators, and be more vigilant about possible related symptoms and signs to detect and handle irAEs as early as possible. For the occurred irAEs, a prompt diagnosis should be made, and they should be graded according to the severity of the disease, followed by symptomatic treatment to relieve the discomfort of patients during the treatment process.

Third, for patients with chronic viral hepatitis, under the condition of strict monitoring of viral hepatitis, the efficacy of ICI treatment is not significantly different from that of uninfected patients, but it is still necessary to closely monitor and handle possible irAEs (32).

Last, the issue of the risk of HBV reactivation must be emphasized during the implementation of immunosuppressive therapy in hepatitis B patients. The APASL Clinical Practice Guideline (47) states that for HBsAg-positive patients, prophylactic antiviral therapy should be initiated immediately prior to the initiation of immunosuppressive therapy and continued until at least 6 to 12 months after the end of therapy. Prophylactic antiviral therapy should also be considered for anti-HBc-positive but HBsAg-negative patients who are receiving high-risk immunosuppressive therapy. Patients not receiving prophylactic antiviral therapy need to be closely monitored for HBV DNA levels and liver function markers, and antiviral therapy should be initiated as soon as HBV reactivation is detected. Therefore, it is indisputable that patients with hepatitis B-related hepatocellular carcinoma implementing immunosuppressive therapy should receive long-term antiviral therapy to avoid HBV reactivation.

In conclusion, although the diagnostic and treatment process of this case has many shortcomings, we hope that the report of this case will raise the awareness of related doctors about ICIs leading to adrenal crisis. When diagnosing and treating hepatocellular carcinoma patients receiving targeted combination immunotherapy, attention should be paid to differentiating septic shock from adrenal crisis to avoid misdiagnosis or omission.

## Data Availability

The raw data supporting the conclusions of this article will be made available by the authors, without undue reservation.
