# Supplementary figures and images for: Adrenal crisis occurring after the application of immune checkpoint inhibitors in a hepatitis B - related hepatocellular carcinoma patient: case report and literature review
Source: Front Immunol. 2025 Nov 27;16:1604740. doi: 10.3389/fimmu.2025.1604740 (PMC12695771; doi:10.3389/fimmu.2025.1604740)

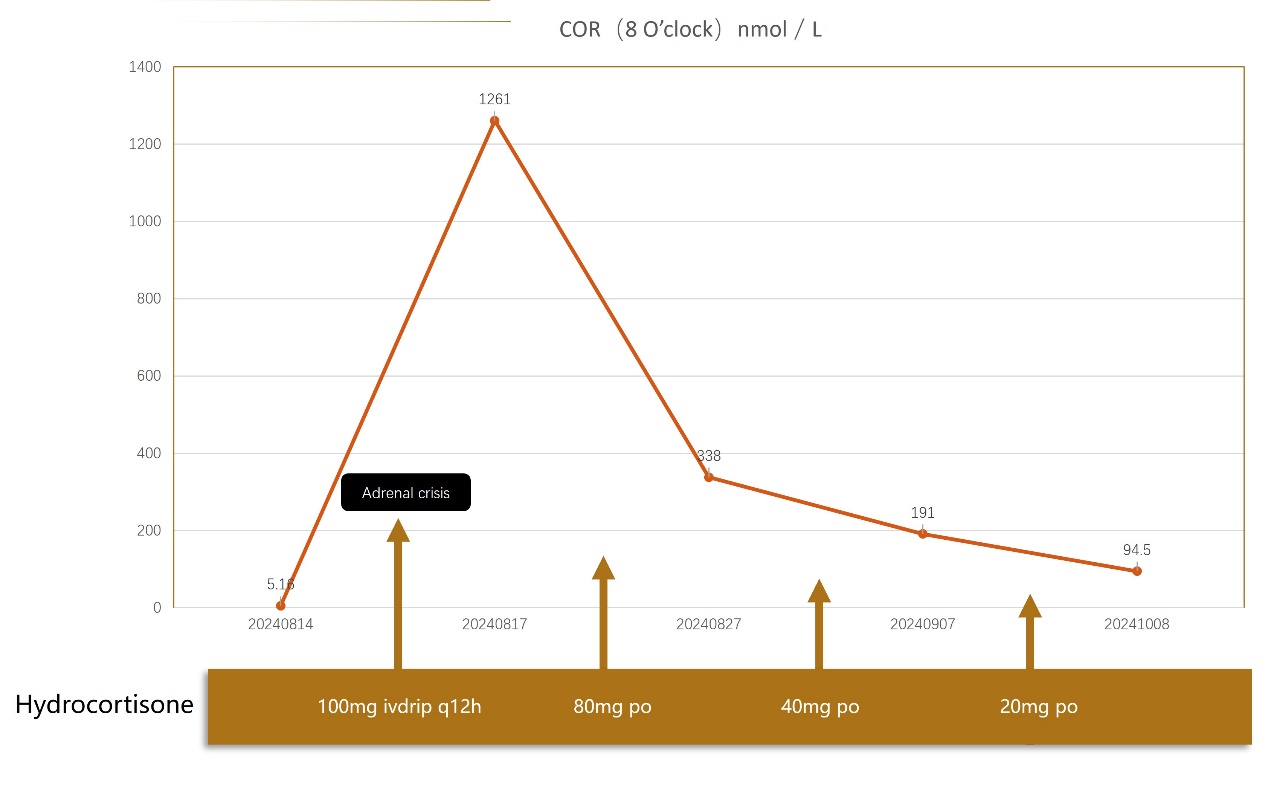


Supplementary Figure 1. Hydrocortisone treatment of the patient and changes in COR levels

Supplement: Supplementary file 1 [file SupplementaryFile1.docx]
